# High-Performance Laterally Oriented Nanowire Solar Cells with Ag Gratings

**DOI:** 10.3390/nano11112807

**Published:** 2021-10-22

**Authors:** Yangan Zhang, Yao Li, Xueguang Yuan, Xin Yan, Xia Zhang

**Affiliations:** State Key Laboratory of Information Photonics and Optical Communications, Beijing University of Posts and Telecommunications, Beijing 100876, China; zhang@bupt.edu.cn (Y.Z.); liyao98@bupt.edu.cn (Y.L.); yuanxg@bupt.edu.cn (X.Y.); xzhang@bupt.edu.cn (X.Z.)

**Keywords:** laterally oriented nanowire, solar cell, Ag gratings, plasmon polaritons, GaAs

## Abstract

A laterally oriented GaAs p-i-n nanowire solar cell with Ag gratings is proposed and studied via coupled three-dimensional optoelectronic simulations. The results show that the gratings significantly enhance the absorption of nanowire for both TM and TE polarized light due to the combined effect of grating diffraction, excitation of plasmon polaritons, and suppression of carrier recombination. At an optimal grating period, the absorption at 650–800 nm, which is an absorption trough for pure nanowire, is substantially enhanced, raising the conversion efficiency from 8.7% to 14.7%. Moreover, the gratings enhance the weak absorption at long wavelengths and extend the absorption cutoff wavelength for ultrathin nanowires, yielding a remarkable efficiency of 13.3% for the NW with a small diameter of 90 nm, 2.6 times that without gratings. This work may pave the way toward the development of ultrathin high-efficiency nanoscale solar cells.

## 1. Introduction

Semiconductor nanowires (NWs) have shown great potential in the field of solar cells thanks to their unique one-dimensional structure and excellent properties including light-trapping, light-concentrating, and effective carrier separation and collection [1,2,3]. Vertically aligned NW array solar cells have long been a research hotpot due to the low filling ratio, high absorption, and the ability to be integrated with heterogeneous substrates [4,5,6,7]. For example, a remarkable efficiency of 15.3% has been obtained for a vertical GaAs NWA solar cell with a low filling ratio of 13% [7]. In recent years, laterally oriented NWs, particularly single NWs, have gained increasing attention [8,9,10,11]. Benefiting from the ultrasmall dimension and ability to be integrated with flexible substrates, single lateral NWs are particularly promising in next-generation miniaturized solar cells for microsystems and wearable devices [12]. So far, single lateral NW solar cells based on different materials and structures have been reported, which have shown great advantages in dimension and flexibility [8,9,12,13,14,15]. Han et al. fabricated a single horizontal GaAs NW Schottky barrier solar cell with an efficiency of 2.8% [12]. Colombo et al. proposed a single lateral p-i-n radial GaAs NW solar cell with an efficiency of 4.5% [16]. Gutsche et al. fabricated a single lateral p-i-n NW diode with a solar conversion efficiency of 4.7% [17]. However, in comparison with vertically aligned NWs and thin films, the efficiency of lateral NWs is relatively low, which is a great challenge in practical applications and in urgent need of improvement.

The low efficiency of lateral NW solar cells is mainly attributed to the relatively poor absorption. Due to the ultrasmall thickness and low filling ratio, the light-trapping and light-concentrating effects in lateral NWs are relatively weak, resulting in strong reflection and low absorption. In addition, the low filling ratio and relatively small refractive index difference between NW and substrate lead to severe absorption of substrate and waste of solar energy. Particularly for ultrathin NWs, the absorption falls precipitously due to the optical diffraction limit. To break through the absorption bottleneck of lateral NWs, the introduction of absorption-enhanced structures is expected to be a feasible and effective approach. However, detailed studies on improvements of lateral NW solar cells are still limited.

In this paper, Ag gratings are introduced to single lateral NW solar cells to enhance the optical absorption. When light is incident perpendicular to the NW, the gratings can generate a propagating wave vector component along the in-plane direction of the NW axis, leading to multiple reabsorption of light by the NW. The structure is built by placing a single lateral axial p-i-n junction GaAs NW on Ag gratings deposited on SiO_2_. GaAs has a desirable direct bandgap of 1.42 eV, which is well matched with the solar spectrum. The light absorption characteristics and photovoltaic performance are studied using the finite-difference time-domain (FDTD) method and finite element method (FEM), respectively. The results show that the gratings significantly enhance the absorption of NW for both TM and TE polarized light, particularly in the 650–800 nm range, which is an absorption trough for pure NW. At an optimal grating period of 400 nm, the short-circuit current is increased from 202 pA to 287 pA, and the conversion efficiency is raised from 8.7% to 14.7%. Moreover, the gratings enhance the weak absorption at long wavelengths and extend the absorption cutoff wavelength for ultrathin NWs, yielding a remarkable efficiency of 13.3% for the NW with a small diameter of 90 nm, 2.58 times that without gratings.

## 2. Methods

**Figure 1:**
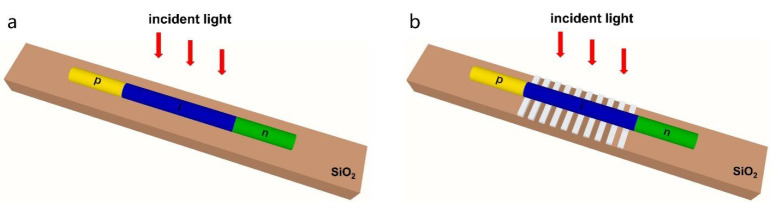
Schematic of laterally oriented NW solar cells without (**a**) and with (**b**) Ag gratings.

Figure 1a,b show the schematic of single lateral NW solar cells without and with Ag gratings, respectively. Both devices contain an 8 μm long axial p-i-n junction GaAs NW placed on SiO_2_, in which the p- and n-regions have an equal length of 2 μm and are uniformly doped to 3 × 10^18^ and 1 × 10^17^ cm^−3^, respectively. The thickness and width of Ag gratings under the i-region of NW are fixed at 100 nm and 450 nm, respectively, while the period is changed to tune the absorption characteristics. Ag gratings can be fabricated on SiO_2_ substrate by photolithography and electron beam evaporation. Axial p-i-n GaAs NWs can be grown by molecular beam epitaxy or metal organic chemical vapor deposition, with Zn and Si as the p- and n-dopants, respectively. As-grown NWs are then transferred onto the gratings by sonication with ethanol as the carrier and aligned in parallel by micromanipulations. Metal electrodes are defined on both ends of NW by photolithography and electromagnetic sputtering or electron beam evaporation.

The performance of the proposed structures is simulated by Sentaurus TCAD (Version H_2013.03, Synopsys, Mountain View, CA, USA). The optical properties are obtained by using the FDTD method to solve Maxwell equations in the Sentaurus Electromagnetic Wave (EMW). The grid used for calculation is generated by the SNMESH. The minimum unit size is 5 nm, and the number of nodes in each wavelength along the *X-*, *Y-*, and *Z*-directions is 10, 10, and 15, respectively. In order to simulate a semi-infinite substrate so that the transmitted light is fully absorbed, a perfectly matched layer (PML) is used near the substrate. The wavelength-dependent complex refractive indices of GaAs, SiO_2_ and Ag can be obtained from [18,19,20]. The infinite extended plane wave is used to simulate the sunlight, whose parameters are derived from a discrete AM 1.5 G solar spectrum. The results of transverse electric (TE) and transverse magnetic (TM) are superimposed to simulate the unpolarized characteristics of sunlight. The total optical generation under the AM 1.5 G illumination can be modeled by superimposing the power-weighted single-wavelength optical generation rates. The optical generation rate $G_{ph}\;$ is obtained from the Poynting vector S.

(1) $$G_{ph}=\frac{\left|\vec{\nabla }\cdot \vec{S}\right|}{2\hbar \omega }=\frac{\varepsilon^{”}{\left|\vec{E}\right|}^{2}}{2\hbar },$$

where ℏ is the reduced Planck’s constant, ω  is the angular frequency of the incident light, E is the electric field intensity at each grid point, and $\varepsilon^{”}\;$ is the imaginary part of the permittivity. The reflection monitor is located above the top surface of the NW, and the transmission monitor is located at the bottom surface to calculate the light absorption. The amount of power transmitted through the power monitors is normalized to the source power at each wavelength. The reflectance R(λ) and transmission T(λ) are calculated using the following equation:

(2) $$R\left(\lambda \right),T\left(\lambda \right)=0.5\int real\left\{p{\left(\lambda \right)}_{monitor}\right\}dS/P_{in}\left(\lambda \right),$$

where P(λ) is the Poynting vector, dS is the surface normal, and $P_{in}\left(\lambda \right)$ is the incident source power at each wavelength. The absorption spectrum A(λ) of the whole structure is given by the following equation:

(3) A(λ)=1−R(λ)−T(λ).

For electrical simulation, the carrier concentration data obtained from the optical simulation is input into the SDevice module, which solves the carrier continuity equations and Poisson’s equation through self-consistent solution to obtain the electrical characteristics, such as the I–V curve. The models mainly include the effect of doping-dependent mobility, carrier radiation composite, Auger composite, and Shockley–Read–Hall (SRH) composite model. The recombination data used in the simulations are obtained from the Levinshtein model [18], which is shown in Table 1. The Arora model is adopted in the calculation of the doping-dependent mobility [21], which is expressed as

(4) $$\mu_{dop}=\mu_{min}+\frac{\mu_{d}}{1+{\left(N/N_{0}\right)}^{A}},$$

where A is 0.6273 (0.8057), and $N_{0}$ is 7.345 × 10^16^ (5.136 × 10^17^) for the electrons (holes).

An optical device consisting of parallel slits of equal width and equal spacing is called a grating. The performance of gratings can be tuned by adjusting parameters, such as the period, groove, the number of slits, and thickness. The basic grating equation can be expressed as

(5) d(sinφ+sinθ)=± kλ, k=1, 2, 3,…,

where d is the grating period, φ is the angle of diffraction, θ is the angle of incidence, λ is the wavelength of the incidence, and k is an integer representing the diffraction order of the grating.

The diffraction order is determined by the grating period and the wavelength of incident light. As has been reported, for a grating period five times as long as the wavelength of incident light, the scalar diffraction theory can be used to conduct correlation analysis, and the incident light will generate multiple orders of diffracted light waves [22]. In that case, when the two are nearly equal, i.e., working under the Bragg condition, only zeroth-order and first^-^order diffraction waves are generated in the diffraction field; hence, it is also called Bragg diffraction. Kogelnik’s two-wave coupling theory can effectively deal with the diffraction characteristics of gratings in this working state [23]. If the grating period is much smaller than the wavelength of the incident light, only zeroth-order diffraction is generated, and the remaining higher-order diffraction waves are all evanescent waves. In this case, the grating is called a subwavelength grating (SWG), and the correlation analysis must be carried out by using vector diffraction theory [24,25]. In general, rigorous coupled wave analysis (RCWA) [26,27] can be adopted. When the incident light is TE polarized light, the diffraction efficiencies of the grating for the *i*-th order reflected diffraction wave and transmitted diffraction wave are as follows:

(6) $$DE_{Ri}=R_{i}R_{i}^{*}Re\left(\frac{k_{1,zi}}{k_{0}n_{1}\cos\theta }\right),$$

(7) $$DE_{Ti}=T_{i}T_{i}^{*}Re\left(\frac{k_{2,zi}}{k_{0}n_{1}\cos\theta }\right).$$

When the incident light is TM polarized light, the diffraction efficiency of reflected diffraction wave is the same as that of TE, and the efficiency of the transmitted diffraction wave is

(8) $$DE_{Ti}=T_{i}T_{i}^{*}Re\left(\frac{\frac{k_{2,zi}}{n_{2}^{2}}}{\frac{k_{0}\cos\theta }{n_{1}}}\right),$$

where $R_{i},T_{i}$ are the normalized electric field amplitude of the *i*-th order reflected and transmitted diffraction wave, $n_{1}$ is the refractive index of air (incident area), $n_{2}$ is the refractive index of the substrate (transmitted region), θ is the angle between the incident light and the normal, $k_{0}$ is the wavenumber of incident light in vacuum, and $k_{1,zi},\;k_{2,zi}$ are the *z* components of the *i*-th order reflected and transmitted diffraction wave number in the incident and transmitted region of the grating.

## 3. Results and Discussion

**Figure 2:**
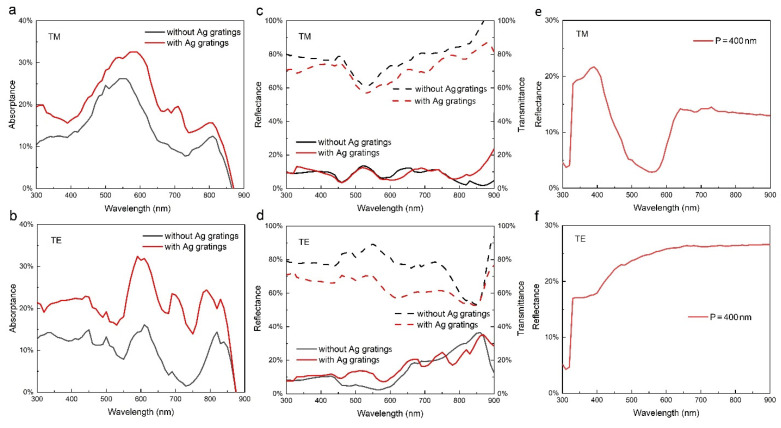
(**a**,**b**) The absorptance of the laterally oriented NW without (black) and with (red) Ag gratings for TM and TE polarized light, respectively; (**c**,**d**) The reflectance (solid line) and transmittance (dotted line) of the laterally oriented NW without (black) and with (red) Ag gratings for TM and TE polarized light, respectively; (**e**,**f**) The reflectance of diffracted light from Ag gratings for TM and TE polarized light, respectively. The grating period and widths are 400 nm and 200 nm, respectively, and the diameter of NW is 180 nm.

The absorptance, reflectance, and transmittance of the laterally oriented NWs for TM and TE polarized light are shown in Figure 2a–d. As the laterally oriented NW is not isotropic in the *x*–*y* plane, the absorption spectra for the TM and TE polarized light are different. Due to the strong transmission and reflection, the absorptance of NW without gratings is generally low. In particular, a distinct absorption trough is observed in the 650–800 nm wavelength range, which severely restricts the conversion efficiency as the sunlight power in this range is relatively high. Therefore, it is of great significance to improve the absorptance at long wavelengths.

According to the theory of diffraction grating, gratings with periods of 300–800 nm are assumed to have excellent performance in the wavelength range of 300–900 nm, and the performance can be modulated by adjusting various parameters. Here, gratings with a moderate period of 400 nm are taken as an example to study the absorption enhancement effect. Figure 2a,b show the absorptance of NWs for both TM and TE polarized light, respectively. It can be seen that, by introducing gratings, both TE and TM absorption is significantly enhanced over the whole wavelength range. The absorption enhancement is mainly the result of a reduction of transmittance, as shown in Figure 2c,d. For TE polarization, the absorption enhancement is attributed to the diffraction of gratings. The gratings generate a propagating wave vector component along the in-plane direction of the NW axis, resulting in multiple reabsorption of light by NW. For TM polarization, the diffraction effect of gratings is weaker, which can be seen from the reflectance of diffracted light for TM and TE polarized light, as shown in Figure 2e,f. Correspondingly, the absorption enhancement for TM polarization is lower than that for TE polarization. Another possible factor contributing to the absorption enhancement for TM polarization is the surface plasmon polaritons (SPPs) [28,29,30]. SPPs can be excited at the interface between air and silver, which propagate along the interface and scatter into free-space mode at the edge of the grating. The scattered light and propagated SPPs can transfer energy to the NW, leading to the light absorption enhancement.

**Figure 3:**
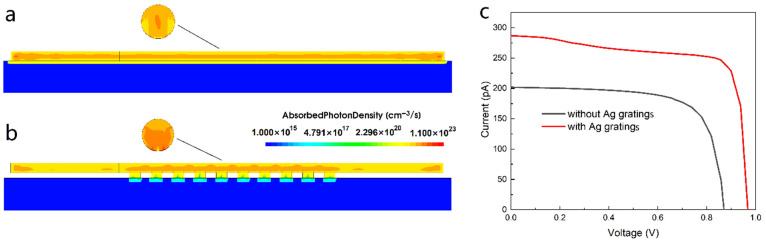
(**a**,**b**) The cross-sectional and longitudinal optical generation profiles of the laterally oriented NW without Ag gratings and with Ag gratings, respectively; (**c**) the current–voltage characteristics of the laterally oriented NW without (black) and with (red) Ag gratings.

To better describe the absorption characteristics of NW, Figure 3a,b show the cross-sectional and longitudinal optical generation profiles of NW without and with gratings, respectively. For NW without gratings, the photocarriers are mainly concentrated at the center of the NW cross-section and distribute uniformly along the whole NW. The photocarriers generated in the highly doped p- and n-regions quickly recombine due to the lack of a built-in electric field, leading to serious recombination loss. Compared with the NW without gratings, the absorption of NW with gratings is significantly enhanced as seen from the cross-section. Moreover, the absorption is mainly concentrated in the middle i-region lying on the gratings, while the absorption in the p- and n-regions is greatly suppressed, which significantly reduces the recombination loss in p-/n-regions. This is because of the concentrating effect caused by the excitation of the local surface plasmons in the metal gratings [29,30,31]. After introducing metal gratings to the i-region, the local optical field is significantly enhanced. The incident light can be coupled to the metal gratings to excite local surface plasmons and transfer the light field energy to NW, thus improving the absorption of incident light significantly. Another phenomenon is that the maximum absorption position moves down toward the NW/grating interface, which can be explained by the diffraction action of the grating redirecting the light to the lower half of the NW without irradiation, leading to a reabsorption of light. On the other hand, the excitation of SPPs also contributes to the transfer of photocarriers to the lower part of the NW. Therefore, by placing the gratings under the i-region of the NW, not only is the total absorption enhanced, but the absorption in highly doped regions is also suppressed, both of which are beneficial for the conversion efficiency improvement of the device.

Figure 3c shows the current–voltage characteristics of NWs without and with Ag gratings, which directly represent the electrical performance of solar cells. After introducing the gratings, the short-circuit current significantly increases from 202 pA to 287 pA, and the open-circuit voltage rises from 0.87 V to 0.968 V, resulting in a remarkable efficiency promotion from 8.71% to 14.7%. The increase in short-circuit current is attributed to the absorption enhancement induced by Ag gratings, as shown in Figure 2. The improvement of the open-circuit voltage is associated with the suppression of recombination loss of carriers [32], as shown in Figure 3b.

**Figure 4:**
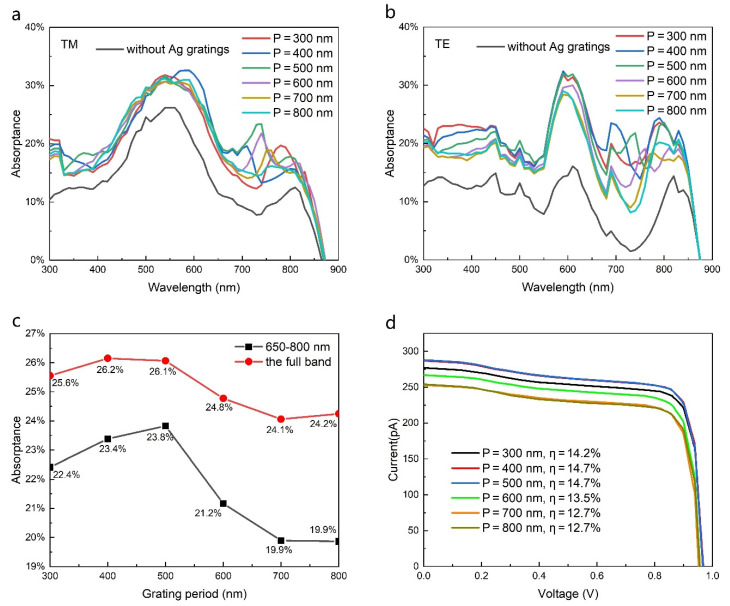
(**a**,**b**) The absorptance of the laterally oriented NWs with different period gratings for TM and TE polarized light, respectively. (**c**) The AM 1.5 G-weighted integral of the NW absorptance with different grating periods. (**d**) The current–voltage characteristics of the NWs with different grating periods. All widths of the grating grooves are half of the grating periods, and the diameter of NWs is 180 nm.

In order to further explore the absorption enhancement mechanism of gratings, the absorptance of NWs with gratings for periods of 300–800 nm was studied, as shown in Figure 4a,b. For TM polarized light, the introduction of gratings generates a new absorption peak at 650–800 nm. With the increase in the grating period, the position of the absorption peak is redshifted, and the value of the peaks first increases and then decreases. This is attributed to the excitation condition of SPP. With the increase in grating period, the excitation wavelength of SPP gradually increases. When the grating period is 500 nm, the new absorption peak value reaches its maximum, and the peak position coincides with that of the absorption trough without gratings. This means that a grating period of 500 nm is optimal for TM polarized light absorption. The reason for this phenomenon is that when the light irradiates the surface of the metal gratings, the incident light interacts with the metal gratings and is coupled to the slit of the gratings, before being coupled to the NW through the slits to enhance its absorption [33]. The wave vector matching condition [34] for exciting the SPPs is

(9) $$\beta =\vec{k}\sin\theta \pm \frac{2n\pi }{d}=\frac{2\pi }{\lambda }\sqrt{\frac{\varepsilon_{d}\varepsilon_{m}}{\varepsilon_{d}+\varepsilon_{m}}}\;,\;n=0,\;1,\;2,\;3,\ldots ,$$

where d is the grating period, β is the propagation constant of SPPs, θ is the angle of the incident light, $\vec{k}$ is the wave vector of the incident light, the air dielectric constant $\varepsilon_{d}$ is 1 C^2^/(N × M^2^), and $\varepsilon_{m}$ is the relative dielectric constant of the Ag, which is related to the wavelength of the incident light.

For TE polarized light, NW with all periods exhibits a larger absorption enhancement compared with that of TM polarization. The absorption enhancement is particularly noticeable in the 650–800 nm wavelength range. As the period varies, the absorption intensity and peak wavelength in this band also change. As mentioned above, the absorption enhancement for TE polarized light is mainly attributed to the diffraction action of the grating, which changes the direction of light propagation and generates a wave vector component along the in-plane direction of the NW axis. According to the grating period and the wavelength of incident light, the absorption enhancement for TE light depends mainly on the action of first-order diffraction light, and the absorption peak wavelength range is redshifted with an increase in the grating period. At periods of 400 nm and 500 nm, the peak wavelength is around 700 nm, matched well with that of the absorption trough without gratings. Taking into account both TM and TE light, the AM 1.5 G-weighted integral of the absorptance of NWs with different grating periods is shown in Figure 4c. In agreement with the above analysis of TM and TE absorption, the total absorptance is optimal at grating periods of 400 and 500 nm. This demonstrates that the absorption enhancement at 650–800 nm plays a dominant role in the performance improvement of laterally aligned GaAs NW solar cells. Figure 4d shows the current–voltage characteristics of NWs with different grating periods. The open-circuit voltage is nearly the same for different grating periods, suggesting that the grating period has little effect on the recombination loss of carriers. The highest efficiency is obtained at grating periods of 400 nm and 500 nm, which is consistent with the absorption results mentioned above. Nevertheless, NW with gratings exhibits higher efficiency in comparison with that without gratings, regardless of the grating period.

**Figure 5:**
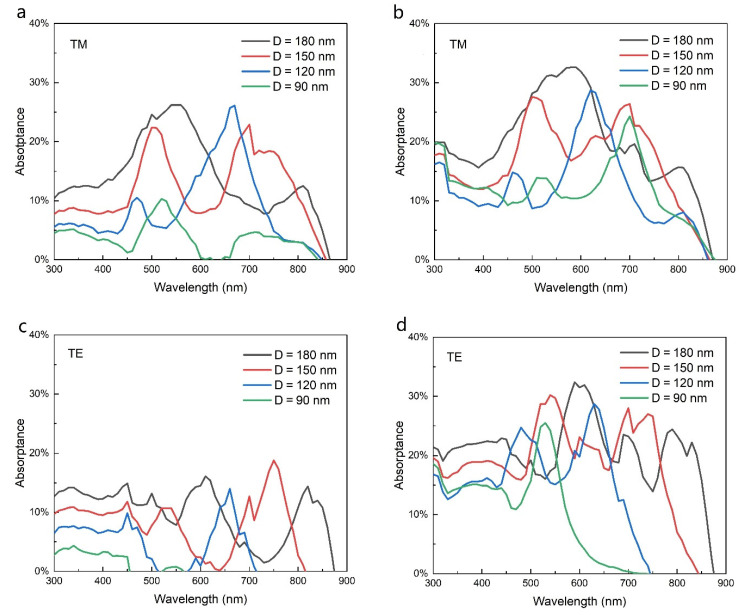
Dependence of the absorptance of NWs on their diameter. (**a**,**b**) Absorptance of TM light for NW without and with gratings, respectively; (**c**,**d**) absorptance of TE light for NW without and with gratings, respectively. The grating period is 400 nm.

Studies have shown that the light absorption of NWs strongly depends on their diameters [10,35,36]. Figure 5 shows the absorptance of NWs with different diameters for TM and TE polarized light. Generally speaking, as the diameter increases, the absorption peak of NWs exhibits a redshift, which is attributed to the optical resonance mode in the NW cavity that causes strong absorption at a specific wavelength [37,38]. However, the absorptance of 90 nm diameter NW does not conform to this rule, which is attributed to the partial loss of optical resonance mode. Figure 5a,b show the diameter-dependent absorptance of NWs without and with Ag gratings for TM polarized light, respectively. As the diameter decreases, the absorption at long wavelength drops, especially in the 700–870 nm range. After the introduction of gratings, the absorption at long wavelengths is improved for NW with all diameters, particularly for an ultrasmall diameter of 90 nm. The excitation of SPPs plays a dominant role in the absorption enhancement of an ultrathin NW, which enables it to support more resonance modes. For TE polarization, due to the diffraction limit of light, an absorption cutoff wavelength is observed for thin NWs without gratings, which exhibits a blueshift as the diameter decreases, as shown in Figure 5c. For the NW with diameter of 90 nm, the absorption cutoff wavelength is about 570 nm, resulting in a huge loss of incident light. After the introduction of gratings, the absorption cutoff wavelength is extended, as shown in Figure 5d. For the NW with a diameter of 90 nm, the cutoff wavelength is significantly extended to 730 nm. This is because the diffraction of gratings significantly improves the confinement of long-wavelength modes for ultrathin NWs [10]. The enhancement of absorption intensity and the expansion of absorption wavelength range induced by gratings are particularly promising for the development of ultrathin high-performance solar cells.

**Figure 6:**
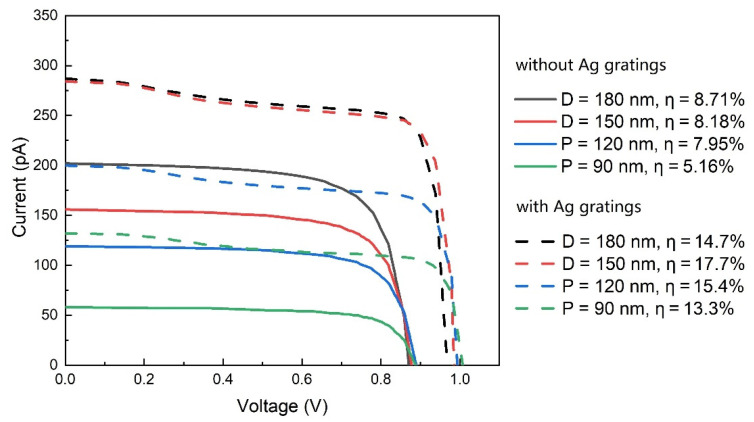
The current–voltage characteristics of NWs with different diameters. The grating period is 400 nm.

Figure 6 shows the current–voltage characteristics of NWs with different diameters. It can be seen that the conversion efficiency is significantly enhanced for NWs with all diameters after the introduction of gratings, and the enhancement increases as the diameter decreases. For example, the efficiency is 1.7 and 2.6 times that of the NW without gratings at diameters of 180 nm and 90 nm, respectively. A remarkable efficiency of 13.3% is obtained on a 90 nm thick NW, which is 1.5 times that of a 180 nm thick one, with a 1/4 material volume. This suggests that the combination of NWs with gratings has great potential in low-cost ultrathin solar cells.

## 4. Conclusions

In summary, a laterally oriented GaAs p-i-n NW solar cell with Ag gratings was proposed and studied. Adding Ag gratings into NWs significantly enhanced the absorption of NW for both TM and TE polarized light due to the combined effect of grating diffraction and the excitation of plasmon polaritons, particularly in the 650–800 nm range, which is an absorption trough for a pure NW. An additional absorption peak near 750 nm was generated after introducing gratings, whose position and intensity changed with the grating period. At an optimal grating period of 400 nm, the recombination loss in highly doped regions was greatly suppressed and the conversion efficiency was raised from 8.7% to 14.7%. Moreover, the gratings enhanced the weak absorption at long wavelengths and extended the absorption cutoff wavelength for ultrathin NWs, yielding a remarkable efficiency of 13.3% for the NW with a small diameter of 90 nm, 2.58 times that without gratings. This work provides an effective avenue for III–V semiconductor materials to achieve ultrathin and high-efficiency solar cells.

## Figures and Tables

**Table 1:** Key material parameters.

Parameters	Electron (Hole)
Minimum mobility	2.136 × 10^3^ (21.48) cm^2^/Vs
SRH lifetime	1 ns
Radiative recombination coefficient	7.2 × 10^−10^ cm^3^/s
Auger recombination coefficient	1.9 × 10^−31^ (1.2 × 10^−31^) cm^6^/s

## Data Availability

Not applicable.
